# Mechanochemical regulation of growth cone motility

**DOI:** 10.3389/fncel.2015.00244

**Published:** 2015-07-07

**Authors:** Patrick C. Kerstein, Robert H. Nichol, Timothy M. Gomez

**Affiliations:** Neuroscience Training Program, Department of Neuroscience, School of Medicine and Public Health, University of Wisconsin-MadisonMadison, WI, USA

**Keywords:** mechanotransduction, axon guidance, TRP channels, substrate rigidity, durotaxis, actin retrograde flow, neuron

## Abstract

Neuronal growth cones are exquisite sensory-motor machines capable of transducing features contacted in their local extracellular environment into guided process extension during development. Extensive research has shown that chemical ligands activate cell surface receptors on growth cones leading to intracellular signals that direct cytoskeletal changes. However, the environment also provides mechanical support for growth cone adhesion and traction forces that stabilize leading edge protrusions. Interestingly, recent work suggests that both the mechanical properties of the environment and mechanical forces generated within growth cones influence axon guidance. In this review we discuss novel molecular mechanisms involved in growth cone force production and detection, and speculate how these processes may be necessary for the development of proper neuronal morphogenesis.

## Biochemical and Mechanical Signal Cross-talk in Growth Cones

The last two decades of intensive research have identified many families of chemical ligands and corresponding receptors that are required for proper neural network assembly during embryonic development (for review, see Chèdotal and Richards, 2010; Kolodkin and Tessier-Lavigne, 2011). Previous studies in several animal models clearly demonstrate that graded chemical ligands deposited in the environment of developing neurons serve as navigational cues that guide neuronal migration and morphogenesis. Soluble, cell surface and substratum-associated extracellular ligands are known to activate receptors linked to numerous intracellular biochemical signal transduction cascades that regulate motility. Most biochemical signals effect cytoskeletal dynamics and membrane trafficking directly, or indirectly through new protein translation, to control growth cone motility (Vitriol and Zheng, 2012; Shigeoka et al., 2013). Receptors on growth cones for growth factors, guidance cues and adhesive ligands activate signals that both promote and inhibit motility. A diverse and complex web of interacting biochemical signals are activated by individual ligands. Signaling is further complicated *in vivo*, where multiple ligands are encountered concurrently and signals are integrated by growth cones and converted into a proper behavioral output (Dudanova and Klein, 2013).

While chemical cues in the environment quite clearly have complex and varied effects on intracellular signaling, new work shows that the mechanical properties of the cellular micro-environment of developing neurons also influence signaling and motility. Immobilized ligands on cell surfaces or secreted into the extracellular environment provide adhesive support for migrating cells. Cell surface receptors physically link to immobilized ligands with varying affinities (Myers et al., 2011; Hynes and Naba, 2012). Molecular adaptor proteins link receptors to rigid cytoskeletal elements that generate opposing forces. Classic studies showed that growing axons produce both contractile (myosin motor driven) and pushing (cytoskeleton polymerization) forces (Letourneau et al., 1987; Lamoureux et al., 1989; Heidemann et al., 1990). These forces are known to reciprocally influence cell signaling as a feedback homeostatic regulator of cell adhesion, shape and movements. Therefore, there is likely complex cross talk between biochemical and mechanical signaling within motile growth cones. In this review, we discuss our current understanding on roles of the mechanical environment and intracellular forces that govern axon guidance.

## Mechanical Force Generation by Growth Cones

**Figure 1:**
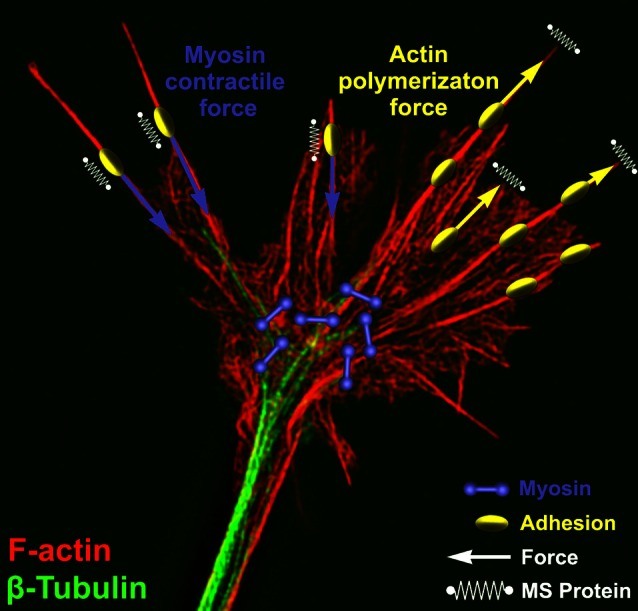
**Force generation and force sensing in neuronal growth cones**. A neuronal growth cone is labeled for filamentous actin (red) and βI-II tubulin (green) using immunocytochemistry. This super resolution image was captured using structure illumination microscopy as previously described (Santiago-Medina et al., 2015). Overlaying the image are schematic elements depicting myosin dimers (purple) and adhesion complexes (yellow) near the central and peripheral growth cone, respectively. Myosin bound to actin produces a rearward force (purple arrows) on adhesion complexes where mechanosensitive (MS) proteins (parallel springs) detect this force. Adhesion complexes antagonize this rearward force allowing actin polymerization to expand the leading edge membrane (yellow arrows) and stretching a set of membrane MS proteins (perpendicular springs).

The cytomechanical forces that control growth cone motility have been intensely studied for the last 30 years, yet our understanding is still incomplete. Similar to non-neuronal cells, actin and microtubule polymerization play central roles as force-generating polymers in axonal growth cones (Figure 1). Complex mechanisms function within growth cones downstream of chemical and mechanical signals to tightly regulate the dynamic assembly and organization of the cytoskeleton (Lowery and van Vactor, 2009; Dent et al., 2011). Leading edge protrusion is thought to be driven largely by F-actin polymerization. Actin polymerization at the leading edge produces tensile forces, which are distributed between plasma membrane protrusion and rearward movement of F-actin bundles (Symons and Mitchison, 1991; Lin et al., 1996; Mogilner and Oster, 2003; Carlier and Pantaloni, 2007). Balance between membrane fluid dynamics and F-actin tensile strength may contribute to the extent of forward protrusion vs. rearward flow of F-actin (Figure 1; Bornschlögl, 2013). Conversely, ADF-cofilin mediated depolymerization of F-actin minus ends relieves compressive actin network forces and replenishes G-actin pools needed for further F-actin polymerization at the leading edge (Bamburg, 1999; Marsick et al., 2010; Zhang et al., 2012). A second force that powers F-actin retrograde flow (RF) is myosin-II motor dimers, which centripetally contract antiparallel F-actin networks toward the growth cone central domain (Turney and Bridgman, 2005; Medeiros et al., 2006; Yang et al., 2013; Shin et al., 2014). The contractile force of myosin-II, coupled with the rearward flow of F-actin due to leading edge polymerization, drives F-actin RF in growth cones (Forscher and Smith, 1988; Lin and Forscher, 1995; Brown and Bridgman, 2003). Other F-actin motor proteins, such as myosin I (Wang et al., 2003), V, VI (Suter et al., 2000; Kubota et al., 2010), and X (Berg and Cheney, 2002), also contribute to growth cone movements, morphology and vesicle trafficking.

During axon development and guidance, the equilibrium between actin polymerization and RF is a key regulator of growth cone protrusion and retraction. Increased leading edge protrusion could theoretically result from either increased actin polymerization or decreased myosin-II contraction. On the other hand, leading edge retraction or collapse could result from reduced actin polymerization, increased depolymerization, or increased myosin-II contraction. Another key force that counteracts RF in growth cones, as in non-neuronal cells (Smilenov et al., 1999; Giannone et al., 2009; Thievessen et al., 2013), are clutching forces at cell-substratum adhesions, which physically link to the F-actin cytoskeleton through a number of adaptor and signaling proteins (Suter et al., 1998; Woo and Gomez, 2006; Bard et al., 2008; Shimada et al., 2008; Myers and Gomez, 2011; Santiago-Medina et al., 2013; Toriyama et al., 2013). The molecular “clutch” is thought to restrain myosin-II mediated contractile forces upon the F-actin network to redirect the force of actin polymerization toward membrane protrusion. Many signaling and adaptor proteins target to growth cone point contact adhesions to regulate clutching, which provide numerous possible sites for regulatory control of axon guidance downstream of soluble, immobilized, and mechanical cues (Bard et al., 2008; Myers and Gomez, 2011; Toriyama et al., 2013).

**Figure 2:**
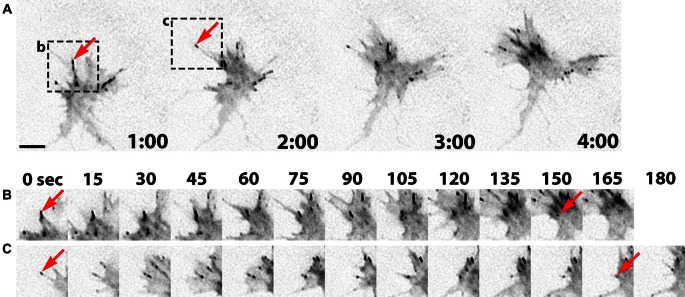
**Point contact adhesion dynamics in neuronal growth cones. (A)** Inverted contrast images of a *Xenopus* growth cone expressing Paxillin-GFP captured every 1 min over a 4 min period using TIRF microscopy. The arrows and black boxes denote the adhesions viewed in **(B)** and **(C). (B,C)** Images of individual adhesions are displayed at 15 s intervals. The arrows indicate the first and last frames of specific paxillin-GFP puncta. This figure was created with an original timelapse captured for demonstration purposes in this manuscript using techniques previously described (Woo et al., 2009; Myers and Gomez, 2011). Scale bar, 5 μm for all panels.

Growth cone point contact adhesions are related to fibroblast focal adhesions, which are multi-functional, macromolecular protein complexes (Smilenov et al., 1999; Bard et al., 2008; Shimada et al., 2008; Geiger et al., 2009; Giannone et al., 2009; Toriyama et al., 2013). However, much less is understood about the molecular regulation and function of growth cone adhesions, and it is likely that these adhesions serve many functions that are unique to growth cones. Point contact adhesions typically assemble within growth cone filopodia that contain parallel bundled actin, have a short lifetime, then disassemble near the base of filopodia (Figure 2). Point contact adhesions appear to require integrin engagement, as they are observed primarily in growth cones on extracellular matrix (ECM) proteins (Woo and Gomez, 2006; Myers and Gomez, 2011). The ECM contains many ligands that modulate growth cone motility, such as laminin, tenascin, fibronectin, etc. Each type of ECM ligand activates specific integrin receptors, as we have recently reviewed (Myers et al., 2011). Activation of integrins leads to recruitment of scaffolding and signaling proteins, such as talin, focal adhesion kinase (FAK), paxillin, zyxin, and α-actinin (Gomez et al., 1996; Cluzel et al., 2005; Robles and Gomez, 2006; Myers et al., 2011). In non-neuronal cells, scaffolding proteins link to actin filaments to clutch RF, which supports actin polymerization to drive protrusion of the leading edge (Smilenov et al., 1999; Giannone et al., 2009; Thievessen et al., 2013). Similar clutching of RF likely occurs at growth cone point contacts (Santiago-Medina et al., 2013) and higher density adhesions have been linked to slower RF (Koch et al., 2012). Importantly, since point contact adhesions are modulated by ECM and soluble guidance factors (Woo and Gomez, 2006; Myers and Gomez, 2011; Myers et al., 2012), it is plausible that growth cone guidance is controlled by local changes in RF. Indeed, early studies from Paul Forscher and colleagues suggested that local reduction in RF is correlated with increased growth cone motility (Lin and Forscher, 1995; Lin et al., 1996; Santiago-Medina et al., 2013). For example, reduced RF is correlated with translocation of the growth cone central domain toward areas of strong adhesion of Aplysia neurons in contact with ApCAM-coated beads (Lin and Forscher, 1995; Suter et al., 1998). While the adapter proteins that link and clutch F-actin differ between cell adhesion molecules, such as ApCAM, L1 and N-cadherin, and integrin-ECM adhesions, they both function to restrain RF and promote axon outgrowth (Lin and Forscher, 1995; Suter et al., 1998; Bard et al., 2008; Toriyama et al., 2013).

The inverse relationship between RF and growth cone motility is well established, however there are exceptions to this model. For example, actin RF and motility both increase in Aplysia growth cones stimulated with 5-HT (5-hydroxytryptamine, serotonin; Zhang et al., 2012). This difference may be stimulus dependent, as 5-HT may increase actin polymerization without modulating adhesion dynamics leading to increase actin drag on existing adhesions. This has been described as the viscous slip clutch model (Giannone et al., 2009; Zhang et al., 2012). Conversely, guidance cues such as brain-derived neurotrophic factor (BDNF) and Semaphorin 3A regulate traction forces and actin RF speeds by changing adhesion dynamics (Woo and Gomez, 2006; Myers and Gomez, 2011). However, it is still unclear whether these two mechanisms operate within individual cells, but work in epithelial cells suggests RF rates may slow at focal adhesions through clutching and increases at the leading edge through increased actin polymerization (Gardel et al., 2008). It remains unclear how clutching mechanisms in growth cones depend upon the adhesive environment, soluble guidance cues and cell type.

**Figure 3:**
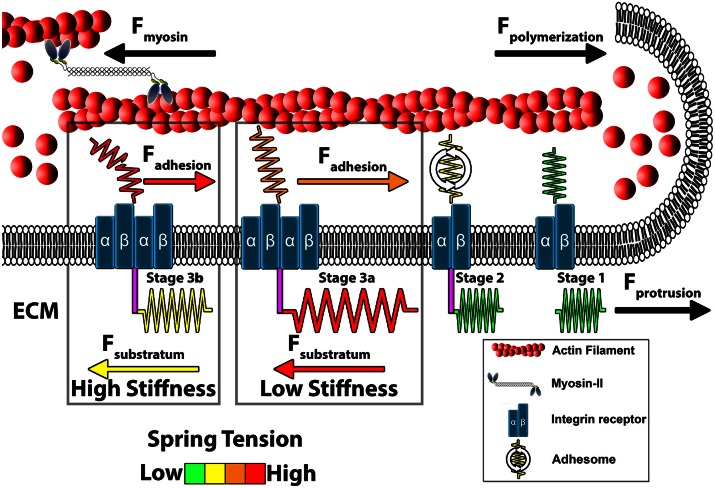
**Model of growth cone traction forces on high and low compliant substrata**. Distal to the leading edge, active myosin-II generates contractile forces (F_myosin_) that pulls F-actin rearward. In addition, actin polymerization at the leading edge pushes against the plasma membrane to propel F-actin rearward (F_polymerization_). These forces integrate to drive constitutive retrograde flow (RF) of F-actin filaments at the leading edge. Stage 1 (ligand unbound). The molecular clutch is disengaged in the absence of integrin activation and clustering leading to rapid RF due to unrestrained F_myosin_ and F_polymerization_. Stage 2 (ligand bound). Upon contact with extracellular matrix (ECM) proteins, integrin receptors become activated, cluster and begin recruiting adhesome-related adaptor and signaling proteins. Stage 3 (clutching). Mature point contact adhesions link with actin filaments (F_adhesion_) to restrict RF and generate traction forces (F_traction_) on the substratum. Therefore, forces generated by clutching of RF are distributed between traction forces with the ECM, adhesive forces on point contacts and protrusive forces at the leading edge. Conditions that maintain clutching of RF produce robust protrusion. Stage 3a (low substratum stiffness). On soft substrata, F_traction_ forces are distributed to the elastic substrata at point contact adhesions through substratum displacement, which reduces F_adhesion_ at point contact adhesions. Lower F_adhesion_ at point contacts prevents clutch slippage (breaking), leading to increased protrusion and growth cone translocation. Stage 3b (high stiffness). Little displacement of the ECM occurs on rigid substrata. Subsequently, most force of RF is transferred to F_adhesion_ at point contacts during clutching. The increased force on adhesions results in breaking or disassembly of point contacts via molecular stretching or activation of cellular signals. Fewer and short lived point contacts on rigid ECM disrupts clutching forces necessary for membrane protrusion and rapid outgrowth.

Increased protrusive forces at the leading edge membrane generated by molecular clutching of F-actin RF, are balanced by adhesive (traction) forces with the cell substratum at adhesion sites (Figure 3). Traction forces with the cell substratum have been measured in migrating cells and growth cones using deformable substrata containing fluorescent tracer beads as fiducial marks (Hyland et al., 2014). Early work showed that cells migrate in the direction of the strongest substratum forces (Lo et al., 2000), which occur at focal adhesions (Plotnikov et al., 2012). In growth cones, these traction forces on the substratum are distributed within the actin-rich peripheral domain, where point contact adhesions are formed (Figure 1; Hyland et al., 2014). In response to guidance cues, localized assembly of adhesion complexes likely yield a redistribution of the traction forces on the substratum. This differential increase in traction forces on one side of the growth cone results in preferential growth in that direction. Moreover, the strength of traction forces generated by cells and growth cones increases on more rigid substrata, suggesting homeostatic regulation of force production (Chan and Odde, 2008; Koch et al., 2012). Substratum elasticity regulates integrin activity, internalization and adhesion site assembly (Du et al., 1993; Friedland et al., 2009), which likely accounts for increased traction forces at higher rigidity. Interestingly, growth cones from different neuronal types have been shown to generate different levels of substratum traction stress. For example, CNS hippocampal neurons exhibit rapid RF rates, due to decreased clutching, and can only generate modest peak traction stress. Conversely, dorsal root ganglion (DRG) neurons, which form more point contact adhesions that slow RF, can generate larger traction forces (Koch et al., 2012). These differences in traction stress may be related to the types of elastic environments CNS vs. PNS neurons encounter.

## Mechanically Sensitive Proteins within Neuronal Growth Cones

**Figure 4:**
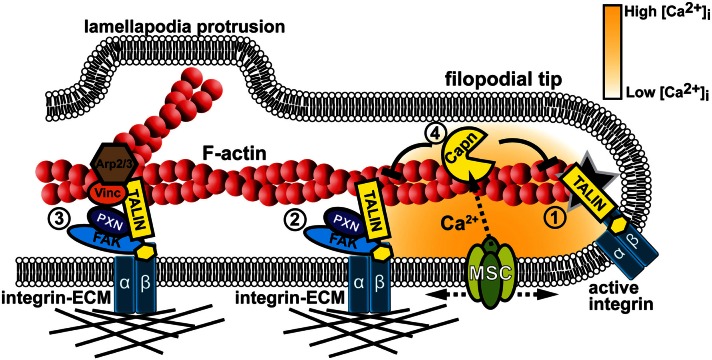
**Mechanotranduction within growth cone filopodia involves adhesion and Ca^2+^ signaling**. (1) Formation of point contact adhesions are initiated by talin-dependent inside-out activation of integrin receptors. (2) Point contact adhesions form when integrins bind to the ECM and intracellular proteins, such as FAK and paxillin, are recruited to signal and link integrins to the actin cytoskeleton. MS proteins, such as FAK, Talin, and CAS (not shown), are activated during substratum rigidity sensing and signaling for adhesion maturation. (3) Additional proteins are recruited during adhesion maturation, such as vinculin and the actin nucleator Arp2/3. Therefore, mature adhesions act as a signaling nexus for new actin filament polymerization off existing filaments, leading to veil protrusion. (4) Adhesion assembly and maturation can be disrupted when Ca^2+^ flows through MS channels (MSC). Ca^2+^ ions directly activate the protease calpain that targets specific adhesion proteins to inhibit or modulate their function (Kerstein et al., 2013).

For cells to sense the mechanical properties of their environment they must express proteins that change their conformation in response to mechanical force or tension. Depending on the type of mechanosensitive (MS) protein, conformational changes may lead to modulation of enzymatic activity, accessibility of binding sites for protein-protein interactions, or regulation of ion channel gating. One site where cell mechanosensors are likely concentrated is at integrin receptor-adhesion protein complexes, which function at the interface between the cytoskeleton and ECM (Figure 4). Adhesion complexes are spatially and temporally regulated by mechanical strain and substrate elasticity (Pelham and Wang, 1997; Schedin and Keely, 2011), suggesting they provide homeostatic feedback, termed tensional homeostasis. In this section we will discuss several recent reports that demonstrate that individual adhesion proteins and MS ion channels can respond to mechanical forces (Table 1).

**Table 1:** **Mechanosensitive (MS) proteins in growth cones**.

Gene name	Mechanical activation	Downstream signaling	Growth cone mechanism	Key references (Bold—Mechano. *Italics—Growth cone*)
**Adhesion proteins**
FAK	Increased kinase activity	• Tyrosine phosphorylation • Regulation of adhesiondynamics	• Required for attractive axon turning • Promotes axon outgrowth	*Robles and Gomez (2006)* *Myers et al. (2011)* ***Moore et al. (2012)***
RPTP-α	Increased phosphataseactivity	• Fyn recruitment to integrins • p130Cas phosphorylation	• Phosphatase activityinhibits axon outgrowth.	***Kostic et al. (2007)***
p130Cas	Increased availability of tyrosine residues	• Phosphorylated by Src and Abl kinases	• Required for *in vivo* and *in vitro* axon pathfinding and dendritic patterning.	**Sawada et al. (2006)** *Huang et al., 2007* *Riccomagno et al. (2014)*
Talin	Increased availability of vinculin binding sites	• Vinculin binding • Reinforcement of integrin -actin linkages	• Required for filopodia and growth cone motility.	**del Rio et al. (2009)** **Margadant et al. (2011)** ***Kerstein et al. (2013)*** *Sydor et al. (1996)*
Filamin	Increased availability of protein binding sites.	• Recruitment of Rho, ROCK, PAK, and PKC to actin cytoskeleton and adhesions.	• Required for *in vivo* axon pathfinding.	**Furuike et al. (2001)** **Razinia et al. (2012)** *Zheng et al. (2011)* *Nakamura et al. (2014)*
**Ion channels**
TRPC1	Channel opening (membrane-stretch/integrin transduction)	• Ca^2+^ influx • Activation of calpain • Activation of calcineurin	• Inhibition of axonoutgrowth • Required for attractive/repulsive axon turning	**Maroto et al. (2005)** *Wang and Poo (2005)* *Shim et al. (2005)* *Wen et al. (2007)* ***Kerstein et al. (2013)***
TRPC5	Channel opening (membrane-stretch)	• Ca^2+^ influx	• Inhibits axon outgrowth	*Greka et al. (2003)* **Gomis et al. (2008)**
TRPC6	Channel opening (membrane-stretch)	• Ca^2+^ influx	• Promotes axon outgrowth • Attractive axon turning	*Li et al. (2005)* **Spassova et al. (2006)**
TRPM7	Channel opening (membrane-stretch/shear stress)	• Ca^2+^ influx • Kinase activity(?)	• Inhibits axon outgrowth	**Oancea et al. (2006)** **Wei et al. (2009)** *Turlova et al. (2014)*
TRPV2	Channel opening (membrane stretch)	• Ca^2+^ influx	• Promotes axon outgrowth	***Shibasaki et al. (2010)***
TRPV4	Channel opening (membrane-stretch/integrin transduction)	• Ca^2+^ influx • Activation of calpain (suggested)	• Inhibits of axon outgrowth	**Matthews et al. (2010)** *Goswami et al. (2010)* ***Kerstein et al. (2013)***
Piezo2	Channel opening (membrane-stretch)	• Ca^2+^ influx	• Unknown	***Coste et al. (2010)***

Several enzymes within adhesion complexes appear to be MS. For example, mechanical forces applied to cells change both the localization and activity of FAK, a key non-receptor tyrosine kinase. Importantly, FAK signaling is necessary for cell behavioral responses to locally applied forces and changes in substratum rigidity (Wang et al., 2001). In growth cones, FAK is essential for point contact adhesion dynamics (assembly/disassembly) and axon guidance (Robles and Gomez, 2006; Woo et al., 2009; Myers and Gomez, 2011) and it is likely that mechanical properties of the environment influence FAK function. For example, FAK activation through deleted in colorectal cancer (DCC) requires both the immobilization of Netrin and acto-myosin contractility, suggesting that the kinase activity of FAK is mechanically dependent. Furthermore, inhibition of FAK during Netrin stimulation disrupts recruitment of adhesion complexes and traction force generation in growth cones (Moore et al., 2012). A second enzyme, receptor-like protein tyrosine phosphatase alpha (RPTP-α), is important for both sensing substratum stiffness and regulating axon extension. RPTP-α co-localizes with αvβ6 integrins where it regulates adhesion signaling by activating Src tyrosine kinases. Specifically on a rigid fibronectin substratum, RPTP-α promotes Src and Cas function and clustering to reinforce adhesion complexes and decrease axon outgrowth of hippocampal neurons (Kostic et al., 2007). However, whether RPTP-α senses substratum stiffness through a direct or indirect mechanism remains unclear.

Scaffolding proteins within adhesion complexes, such as p130Cas, talin, and filamin, also likely act as mechanosensors. Stretching of p130Cas exposes cryptic tyrosine residues that are phosphorylated by Src and Abl kinases and initiate several signaling cascades (Sawada et al., 2006). Loss of p130Cas function in developing neurons leads to aberrant axon pathfinding and dendrite patterning *in vivo* (Huang et al., 2007; Riccomagno et al., 2014). Recent evidence suggest that p130Cas may also be regulated by mechanically-dependent Netrin-DCC signaling during axon guidance (Moore et al., 2012). Similarly, stretching of talin leads to increased binding of the adapter vinculin to reinforce integrin-actin linkages (del Rio et al., 2009; Margadant et al., 2011). Talin has bifunctional roles in growth cones where it is important for the assembly of adhesions and as an integral scaffold within point contact adhesions (Sydor et al., 1996; Kerstein et al., 2013). Disruption of talin function leads to changes in filopodial dynamics and reduced growth cone motility (Sydor et al., 1996; Kerstein et al., 2013). In a similar fashion, filamins are scaffolding proteins that are stretched when bound between integrins and F-actin. Tension along filamin unveils cryptic binding sites for many signaling molecules such as RhoA, Rho-associated coiled-coil kinase (ROCK), p21-activated kinase (PAK), and PKC (Furuike et al., 2001; Razinia et al., 2012). In developing animals, mutations in filamins produce premature axon termination, ectopic branching, and aberrant pathfinding *in vivo* (Zheng et al., 2011; Nakamura et al., 2014).

A second set of mechanosensors in growth cones are MS ion channels (Table 1). MS channels have been most well studied in hair cells of the auditory system and the primary afferents of the somatosensory system (Geffeney and Goodman, 2012). However, these channels also have important roles in cell motility. MS channels regulate cell migration and growth cone motility through direct control of Ca^2+^ signaling (Lee et al., 1999; Jacques-Fricke et al., 2006; Franze et al., 2009; Wei et al., 2009; Kerstein et al., 2013). Evidence suggest that cell-substratum interactions activate MS channels in motile cells since Ca^2+^ signals localize to adhesion clusters and in areas of high traction forces (Figure 4; Gomez et al., 2001; Doyle et al., 2004; Franze et al., 2009). In addition, substratum stiffness modulates Ca^2+^ influx as rigid substrata elicit a higher frequency of Ca^2+^ transients, which inhibits axon extension (Kim et al., 2009; Kerstein et al., 2013). Pharmacological inhibitors of MS channels have strong effects on growth cone motility. MS channel inhibitors such as, GsMTx4, Gd^3+^, Gentamicin all reduce the frequency of Ca^2+^ transients and accelerate axon extension (Jacques-Fricke et al., 2006; Kerstein et al., 2013). Furthermore, focal force applied to a growth cone induces collapse and retraction of the axon, which is blocked by inhibition of MS channels (Franze et al., 2009).

Recent reports suggest that growth cones express several types of MS ion channels, such as members of the Transient Receptor Potential (TRP) family. TRP channels form both homo- and heterotetrameric cation channels. Increasing evidence suggest an important role for TRP channels for environmental sensing from entire organisms to single cells. TRP channels transduce extracellular stimuli, like mechanical force, into biochemical signals through the influx of the second messenger Ca^2+^ (Wu et al., 2010). Interestingly, several TRP channel subunits that are expressed in developing neurons are believed to be MS, therefore these MS channels may also be important for axon guidance. For example, TRPV2 can be activated by membrane stretch of developing neurons and expression of dominant negative TRPV2 in developing primary motor neurons inhibits axon extension *in vivo* (Shibasaki et al., 2010). However it is unclear how TRPV2 is mechanically activated *in vivo*. One possibility is that substratum rigidity modulates MS channel activity to regulate growth cone motility. Local filopodial Ca^2+^ transients appear to be generated by mechanical activation of transient receptor potential channel 1 (TRPC1), since these signals are reduced when rigidity of the substratum is decreased. In addition, the effects of MS channel blockers on axon outgrowth is lost with knockdown of TRPC1 and partially lost with knockdown of TRPV4 (Kerstein et al., 2013). Importantly, previous findings suggest that TRPC1 is an essential channel for axon pathfinding *in vitro* and *in vivo* (Shim et al., 2005; Wang and Poo, 2005; Wen et al., 2007). Other TRPC channels are also important during axon guidance and have been implicated in cellular mechanotransduction. TRPC5 is activated by hypo-osmotic stimulation and membrane stretch, and inhibited by the MS channel blocker GsMTx4 (Gomis et al., 2008). TRPC5 also plays a critical role in neurite initiation, axogensis, and axon extension (Greka et al., 2003; Hui et al., 2006; Wu et al., 2007; Davare et al., 2009). Similarly, TRPC6 is activated by membrane stretch and inhibited by GsMTx4 (Spassova et al., 2006) and is also important for axon growth and guidance in response to attractive guidance cues (Li et al., 2005). Recent evidence showed an important role for TRPM7 in the regulation on hippocampal axon extension *in vitro* (Turlova et al., 2014). In addition, TRPM7 was previously shown to respond to membrane stretch and fluid sheer force (Oancea et al., 2006; Wei et al., 2009). A major open question in the field is why are so many TRP channels mechanically sensitive. An intriguing new theory suggests that many TRP channels share a gating mechanism that relies on the local tension and shape of the plasma membrane (Liu and Montell, 2015). Furthermore each MS-TRP channel may be specific for a single neuron type. For example, the expression profiles of TRPC subfamily alone vary widely within the nervous system and exhibit even greater variation during development (Riccio et al., 2002; Strübing et al., 2003; Von Niederhäusern et al., 2013). One final possibility is that only a single TRP channel subunit is mechanically sensitive, but it may form tetrameric channels with many different TRP subunits depending on the cell type. This could result in many different channel tetramers being mechanically sensitive, with contribution from one subunit being sufficient to form a MS channel.

MS ion channels outside the TRP channel family may also be important. Piezo (Fam38a/b) ion channels were identified as MS channels in a siRNA based screen in the mechanically excitable Neuro2D cell line (Coste et al., 2010). Piezo1 controls cell migration in non-neuronal cells, possibly through regulating integrin binding activity (McHugh et al., 2010, 2012). However, Piezo1 is mainly expressed in non-neuronal cells, but Piezo2 is expressed neurons and is essential for the sense of touch in vertebrates (Faucherre et al., 2013; Ranade et al., 2014; Woo et al., 2014). Currently our understanding of Piezo2 is limited to adult animals, so future studies will need to address whether this MS channel has a role in neuronal development and growth cone motility.

How mechanical forces are transduced into MS channel gating is uncertain, but may involve coupling with the cytoskeleton (Clark et al., 2007; Hayakawa et al., 2008) interactions with lipids (Anishkin et al., 2014) and second messenger signals (Vriens et al., 2004; Alessandri-Haber et al., 2008). One intriguing site where local mechanical forces may regulate the opening of MS channels is at cell-cell contact sites or integrin adhesions with the ECM (Hayakawa et al., 2008; Kobayashi and Sokabe, 2010; Kazmierczak and Müller, 2012; Eisenhoffer and Rosenblatt, 2013). In support of this notion, both Ca^2+^ signals and TRP channels localize near integrin adhesion sites (Gomez et al., 2001; Matthews et al., 2010; Kerstein et al., 2013). In neurons, these Ca^2+^ signals may act as a feedback mechanism on adhesion assembly and disassembly controlling growth cone motility (Robles et al., 2003; Kerstein et al., 2013). Interestingly, mechanical gating of MS channels likely depends on substratum rigidity and traction forces (Doyle et al., 2004; Munevar et al., 2004; Kerstein et al., 2013). This suggests that differences in the elastic environment of neurons may control their development *in vivo* through regulation of MS channel expression and function. In addition, MS channels may exert homeostatic regulation of the cytoskeleton and adhesion complexes through activation of downstream Ca^2+^ effectors. Previous studies have identified the Ca^2+^ effectors, calcineurin, CaMKII, and calpain as the main signaling pathways that regulate Ca^2+^ dependent growth cone motility and axon guidance (Robles et al., 2003; Wen et al., 2004). However the most intriguing example of mechanical feedback is the Ca^2+^-dependent protease calpain since it cleaves several adhesion and actin binding proteins to inhibit or modulate their function (Figure 4; Franco and Huttenlocher, 2005). Recent studies have shown that axon outgrowth and morphology are regulated through calpain specific cleavage of talin and cortactin, respectively (Mingorance-Le Meur and O’connor, 2009; Kerstein et al., 2013). However, additional calpain targets in growth cones are likely to exist. For example, in non-neuronal cells calpain cleaves the adhesion proteins Src, FAK, and Paxillin and the actin binding proteins Filamin and α-actinin (reviewed in Franco and Huttenlocher, 2005; Chan et al., 2010; Cortesio et al., 2011). It is important to further elucidate the mechanism of feedback inhibition between MS channels and adhesion/cytoskeleton structures particularly in neuronal growth cones.

An interesting area where mechanotransduction has not been studied in growth cones is membrane trafficking. Endocytosis and exocytosis play an important role in growth cone navigation. For example, endocytosis of membrane and surface integrin receptors is required for repulsive growth turning from myelin-associated glycoprotein and semaphorin 3A (Hines et al., 2010; Tojima et al., 2010). Conversely, exocytosis is important for attractive axon turning and increased axon branching (Tojima et al., 2007, 2014; Winkle et al., 2014). Endocytosis and exocytosis may also directly influence mechanical signals. Recent studies showed that during cell migration leading edge protrusions increase plasma membrane tension, which activates further exocytosis to relieve increased tension (Gauthier et al., 2011). However, the mechanism of sensing membrane tension remains unknown. One possibility is that membrane tension activates MS ion channels. Consistent with this, hypotonic solutions induce cell swelling and increase membrane tension, which activates Ca^2+^ influx through MS channels in growth cones (Jacques-Fricke et al., 2006; Kerstein et al., 2013). Another possibility is that membrane tension is required for the localization of leading edge signaling proteins such as the GTPase Rac1 and the SCAR/WAVE complex, as shown recently in neutrophils (Houk et al., 2012). Unfortunately, our understanding of the relationship membrane tension and trafficking is limited in neuronal growth cones and a considerable amount of work remains before a mechanism is completely understood.

## Mechanical Properties of the Environment Regulate Cell Migration and Axon Growth

Pioneering studies on the mechanisms of growth cone guidance performed both *in vitro* and *in vivo* focused on the role of cell adhesion as a principal determinant of pathfinding (Gomez et al., 1996; Woo and Gomez, 2006; Bard et al., 2008; Bechara et al., 2008; Myers et al., 2011). Using patterned substrata of differential adhesivity, investigators showed that growth cones could be directed *in vitro* simply by differences in adhesion (Hammarback et al., 1985; Gomez and Letourneau, 1994). These early experiments implied that axons may be targeted *in vivo* by adhesive interactions with extracellular ligands. A number of different cell recognition molecules on the surface of growth cones are known to have adhesive properties (Rutishauser, 1985; Lagenaur and Lemmon, 1987; Schmidt et al., 1995), which function to stabilize leading edge protrusions. While it is still uncertain the relative contributions of adhesion vs. biochemical signaling in the control of axon pathfinding *in vivo*, it is likely that differential cell adhesion has some influence (Caudy and Bentley, 1986a,b; O’Connor et al., 1990).

In addition to the classical ECM components, axon guidance cues are immobilized in the extracellular environment to some extent. While this includes large ECM proteins and cell surface adhesion molecules, small secreted growth factors and chemokines are also likely immobilized. Growing evidence suggests that growth factors bind with high specificity to fibronectin type III repeats and heparin sulfate glycosaminoglycans contained within many ECM proteins such as fibronectin, tenascin, and laminin (Hynes, 2002, 2009). Growth factor binding to the ECM likely serves to localize or concentrate soluble factors [e.g., fibroblast growth factor (FGF), Wnt, bone morphogenic proteins (BMPs)] near the cell binding sites and help establish stable gradients necessary for pattern formation *in vivo*. In some cases, growth factor receptors may cooperate with cell adhesion receptors (e.g., integrin) for cell binding, as has been demonstrated for a5ß1 and vascular endothelial growth factor (VEGF) receptor (Rahman et al., 2005). Secreted axon guidance cues have also been demonstrated to bind ECM proteins suggesting that immobilized guidance factors serve as adhesive ligands or provide mechanical support in association with ECM. For example, the repellant Slit must bind collagen for proper lamination of the zebrafish optic tectum (Xiao et al., 2011). Other secreted axon guidance cues such as netrins, BMPs and Sema3s bind the ECM and induce mechanical signals (Hu, 2001; Manitt and Kennedy, 2002; De Wit et al., 2005; Moore et al., 2012). To provide an additional level of control, local proteolysis of ECM may serve to release growth factors in a spatially and temporally controlled manner. Growth cones have recently been shown to target matrix metalloproteases using invadosomes (Santiago-Medina et al., 2015). Therefore, while bath application or local gradients of soluble guidance cues has been the prevailing method for studying axon guidance behaviors *in vitro*, the role of mechanical signaling by immobilized ligands in three dimensional environments is an important and often overlooked consideration for our full understanding of the mechanisms of neural network formation.

Growth cones *in vitro* have been shown to generate tension on neighboring cells and the underlying matrix (Lamoureux et al., 1989; Balgude et al., 2001; Moore et al., 2010; Moore and Sheetz, 2011). Many axon guidance cues are immobilized in the extracellular environment to some extent (as described above), therefore mechanical tensile forces likely contribute to many attractive and repulsive (release of adhesion) guidance behaviors. Recent evidence suggest that even under classic chemical gradient turning assays, mechanotransduction is necessary for chemoattraction. For example, chemical gradients of netrin-1 generated *in vitro* only promote growth cone turning when netrin can bind to the substratum (Moore et al., 2009). Here growth cones utilize netrin as an adhesive ligand that supports traction forces that exceed 60 pN (Moore et al., 2009). Inhibition of netrin substrata adsorption, via co-treatment with heparin, inhibited axon outgrowth (Moore et al., 2012). Interestingly, commissural interneurons *in vivo* will turn towards ectopically secreted full length netrin, but are not guided towards truncated netrin lacking the domains required for substratum adsorption (Moore et al., 2012). Further, guidance toward Netrin depends upon FAK activity, a MS kinase, and myosin-II motor-induced traction forces. Other “soluble” axon guidance cues may require immobilization to elicit their guidance effects on extending axons. For example, ephrinA5 appears to produce repulsive turning or collapse in both border turning assays or as a soluble ligand, but the ability of “soluble” ephrinA5 to bind was not tested in this study (Weinl et al., 2003). EphrinA repellants may disrupt growth cone adhesion to the ECM or to neighboring cells by preventing point contact formation (Woo et al., 2009), or by activating metalloprotease-mediated cleavage of cell surface ephrinA ligands (Hattori et al., 2000), respectively.

While immobilized ligands clearly support traction forces generated by cells and growth cones, many recent studies show that substratum elasticity, or stiffness, also influences the development of neurons (Tyler, 2012; Franze, 2013). Cells and neurons have a remarkable ability to adapt to their mechanical environment. While most cells minimally require anchorage to a solid substratum and do not fully differentiate in liquid suspension, the elasticity of the supporting adhesive substratum can vary over a wide range. Neurons are particularly adaptable cells, as they will morphologically differentiate on extremely soft substrata and tissues, such as brain [Young’s modulus = ~100–1000 Pascal (Pa)], as well as on extremely rigid environments, such as ECM-coated glass and bone (>10 gPa, 20 mPa, respectively), covering an impressive >10, 000 fold range of elasticities (Kruse et al., 2008; Tyler, 2012). Importantly, the elastic modulus (rigidity) of the cell substratum can strongly influence cell differentiation, morphology, motility, and survival (Geiger et al., 2001, 2009; Moore and Sheetz, 2011; Musah et al., 2014). Understanding the roles of substratum elasticity on neuronal development is critical since developing axons and dendrites will encounter widely varying elastic environments during pathfinding to their targets.

Numerous studies over the past decade have shown that growth cone motility depends on the compliance of the cell substratum (Tyler, 2012; Franze, 2013). This is a cell migration process known as *durotaxis*. Previous studies used a variety of materials to generate variable elastic cell culture conditions to study neuronal durotaxis, including polyacrylamide (PAA; Flanagan et al., 2002; Georges et al., 2006; Kostic et al., 2007; Jiang et al., 2008; Koch et al., 2012) and polydimethylsiloxane (PDMS) in 2D (von Philipsborn et al., 2006; Cheng et al., 2011; Kerstein et al., 2013), as well as collagen I gels (Willits and Skornia, 2004; Sundararaghavan et al., 2009) and agarose hydrogels for 3D conditions (Hammarback and Letourneau, 1986; Balgude et al., 2001; Mai et al., 2009). Here we compare results across these conditions (Table 2), but it is important to note that many variables beyond substratum compliance may contribute to observed differences. For example, non-biological materials typically must be conditioned with a biological ligand to support axon extension. A range of ECM proteins at varying concentrations or serum have been used to promote axon outgrowth. In addition to the variable biological conditions tested, the methods used to measure the elastic moduli of the cell substrata also varies. Therefore, Young’s modulus values determined by atomic force microscopy (AFM) may differ from those measured by sheer stress rheometry or micro-position displacement devices. These caveats should be considered whenever comparing between different studies.

**Table 2:** **The effects of substrate rigidity on neurite outgrowth and morphology**.

Neuron type	Substrate (ECM)	Elasticity range (Modulus)	Neurite phenotype	Reference
E9 Chick DRG	3D Agarose (None)	0.003–0.130 kPa* (Shear)	Increased length on soft substrates	Balgude et al. (2001)
E9 Chick DRG	3D Collagen I Gel (varied collegan conc.)	0.002–0.017 kPa (Shear)	Increased length on soft substrates	Willits and Skornia (2004)
E8 Chick DRG	3D Collagen I Gel (varied genipin crosslinking)	0.05–0.80 kPa (Shear)	Increased length on soft substrates	Sundararaghavan et al. (2009)
E13.5 Mouse Spinal Cord	PAA (Matrigel)	0.050–0.550 kPa* (Shear)	Increased branching on soft substrates	Flanagan et al. (2002)

P0 Mouse Hippocampal	PAA (Fibronectin or Laminin)	0.5–7.5 kPa (Young’s)	Increased length on soft substrates	Kostic et al. (2007)
P0 Rat DRG	PAA (Laminin)	0.150–5.0 kPa (Young’s)	Maximum length on 1.0 kPa substratum.	Koch et al. (2012)

E18 Rat Hippocampal	PAA (Laminin)	0.150–5.0 kPa (Young’s)	No affect on length	Koch et al. (2012)
E16 Rat Spinal Cord	PAA (PDL or Collagen I) (DNA oligonucleotide pairs used to vary crosslinking)	6.6–30 kPa (Young’s)	Increased length on soft substrates.	Jiang et al. (2008)
Adult Mouse DRG	PDMS (Poly-L-Lysine)	18–1882 kPa (Young’s)	Maximum length on 88 kPa substratum	Cheng et al. (2011)
Stage 22 Xenopus spinal cord	PDMS (Fibronectin)	950–1800 kPa (Young’s)	Increased outgrowth on soft substrates	Kerstein et al. (2013)
E8–9 Chick DRG	Silk Fibroin Hydrogel (Fibronectin or Laminin)	4–33 kPa (Young’s)	Maximum length on 7–22 kPa substrates	Hopkins et al. (2013)

Abbreviations: kPa, kiloPascals; PAA, polyacrylamide; PDMS, Polydimethylsiloxane. *kPa were converted from kdynes/cm^2^.

One of the first studies to examine neurite outgrowth under different elastic conditions cultured dorsal root ganglia (DRG) neurons within varying concentrations of agarose gels in the presence of 10% fetal bovine serum (FBS). This study found that axons extend more rapidly within softer gels (<20 Pa) and that the rate of outgrowth plateaus above 60 Pa (Balgude et al., 2001). More recently, PAA gels were used to test the effects of substratum elasticity on DRG axon outgrowth. By examining neurite lengths from fixed DRG neurons plated on PAA coated with laminin, Jeff Urbach and colleagues found that axon outgrowth peaks on 1000 Pa gels and decreases on PAA gels below and above this optimal elasticity (Koch et al., 2012). Interestingly, under the same conditions, this group found that hippocampal neurons exhibit no preference for soft substrata, suggesting this behavior may be selective for peripheral neurons. However, this result is contradictory to a previous report that found that hippocampal axons preferred soft conditions (500 Pa) over more rigid (4 KPa; Kostic et al., 2007). It is important to note that the latter study differed from the former as they used PAA gels coated with fibronectin rather than laminin. Other studies using different types of neurons and culture conditions, including within collagen gels, have found preferential outgrowth on softer substrata (Table 2, Balgude et al., 2001; Flanagan et al., 2002; Willits and Skornia, 2004; Georges et al., 2006; Jiang et al., 2008; Kerstein et al., 2013), but it will be important to standardize experimental conditions and elastic modulus measurements to make general statements regarding the effects of the mechanical environment on outgrowth. In addition to effects on axon outgrowth, other studies have shown that softer substrata promote neurite branching of several neuronal types (Wang et al., 2001; Flanagan et al., 2002; Georges et al., 2006; von Philipsborn et al., 2006).

## Mechanical Environment Influences Cell Migration and Axon Growth *In vivo*

Growth cone navigation may be influenced by surrounding physical barriers causing them to grow around or in between different tissue structures. For example, when a severed motor neuron axon begins to regenerate it typically will extend, branch, and synapse in the same location as the pioneer axon. Regenerating axons are physically constrained to the tubes that Schwann cells formed around the original axon (Nguyen et al., 2002). Interestingly, peripheral axon regeneration declines with age, not because the axons lose their intrinsic outgrowth capabilities, but due to the lack of clearance of glial and nerve debris that impose physical barriers to the regenerating axons (Kang and Lichtman, 2013). In the central nervous system (CNS), similar regeneration limitations have been observed when immature neurons were transplanted into rats with striatal lesions (Isacson et al., 1995). In addition to regenerating axons, physical interactions are important during development as well. For example, zebrafish lateral line axons extend with the collective migration of lateral line primordial cells through a physical, but not chemical, interaction. The authors described this as a “tugging” action on the axons by the migrating lateral line primordia (Gilmour et al., 2004). Furthermore, zebrafish Rohan-Beard peripheral axons normally project ventrally, however when the contractions of the underlying muscle tissue are prevented, genetically or pharmacologically, these axons project more longitudinally (Paulus et al., 2009). In Zebrafish *diwanka* mutants, MN growth cones fail to exit the spinal cord into the periphery (Granato et al., 1996; Zeller and Granato, 1999; Schneider and Granato, 2006). *diwanka* mutations were found to be in a lysyl hydroxylase protein (LH3), an enzyme with glycosyltransferase activity that modifies type XVIII collagen. Interestingly, the glycosyltransferase activity of LH3 functions within adaxial cells to chemically modify collagen XVIII that is deposited on the surface of the developing myotome. In this model, collagen XVIII is glycosylated by LH3 and secreted into the ECM where it becomes a suitable substratum to promote the exit of motor axons into the periphery. While it is unclear what the precise roles of collagen glycosylation are toward developing axons, there is evidence it can regulate fibrillogenesis, crosslinking, remodeling and collagen–cell interactions, all of which likely influence mechanical signaling (Yamauchi and Sricholpech, 2012). Glycosylation of dystroglycan also regulates axon guidance at the floor plate of the developing mouse spinal cord and is required for binding and localizing Slit to the floor plate (Wright et al., 2012). However, it is unknown whether the immobilization of Slit is required full receptor activation, as it is for the ligand Netrin (described above). It will be important in the future to determine which immobilized axon guidance cues, such as ECM, cell adhesion molecules and growth factors, use mechanical signaling to elicit their effects on growth cones.

## Axons Extend Through a Mechanically Diverse Environments *In vivo*

The elasticity of the extracellular environment *in vivo* varies across tissues, with age and under pathological conditions. For example, in breast cancer tissues, mammary carcinoma cell migration is influenced by the physical properties of the collagen matrix, such as fiber alignment, density, and stiffness (reviewed in Schedin and Keely, 2011). Interestingly, increases in density and stiffness of the ECM within breast tissue and mammary gland tumors are the key risk factors for the development of breast cancer and the metastatic potential of cancer cells (Boyd et al., 1998; Paszek et al., 2005; Provenzano et al., 2009). These findings under pathological conditions suggest that normal cell migration and the extension of neuronal processes, may also be influenced by local differences in the mechanical environment *in vivo*. The environment of elongating axons and dendrites, which can traverse great distances, varies widely in substratum elasticity (Young’s Modulus) from flexible neural tissues to highly rigid skin, muscle and bone suggesting a role for growth cone durotaxis *in vivo*. (Discher et al., 2005, 2009). Furthermore, local variations in substratum elasticity have been observed within the CNS. AFM measurements made from dissociated hippocampal and retinal neurons found that individual neurons were more rigid than glial cells (Lu et al., 2006). Therefore, the extension of axons and dendrites along different cell types may be influenced by the intrinsic mechanical properties of the cells. In addition, regional differences in tissue elasticities have been observed across specific regions of the CNS. Scanning force micrographs of rat cerebellar slices showed that gray matter is more elastic than white matter (Christ et al., 2010). In addition, slices from developing mouse cerebral cortex show variations in tissue elasticity in both a spatial and temporal manner, suggesting a mechanical niche may influence cellular differentiation, migration, and morphology *in vivo* (Iwashita et al., 2014).

Substratum elasticity *in vivo* is determined by composition, density and orientation of different ECM components. For example, recent work has shown that cross-linking of collagen fibers by lysyl oxidase (Lox) in the drosophila eye disc controls the stiffness of the tissue. Further, disruption of Lox leads to ectopic migration of glial cells suggesting tissue stiffness defines specific migration patterns (Kim et al., 2014). In addition, Lox knockdown or overexpression in the mouse cerebellum modulates Purkinje neuron dendritic development, where increased Lox activity may inhibit outgrowth through increasing collagen cross-linking and substratum stiffness (Li et al., 2010). Recent studies have provided evidence to support a role for substratum stiffness in guiding developing axons *in vivo*, but future studies will need to clarify how and when axon growth is specifically modulated by substratum elasticity. In addition, the study of mechanosignaling during axon guidance requires the development of a robust and reliable *in vivo* model of growth cone durotaxis.

## Future Prospectives and Challenges to the Field

There are many outstanding questions regarding the roles of mechanical forces in the regulation of growth cone motility and axon guidance. Within cells and growth cones, it is unclear which proteins function as mechanosensors and how molecular forces are transferred onto target proteins. In particular, it is not clear how MS ion channels are gated at the plasma membrane and the molecular targets of calcium signals. Ion channel gating may occur at point contact adhesions, where myosin II contractile forces are focused at integrin-ECM contacts (Gomez et al., 2001; Hayakawa et al., 2008; Matthews et al., 2010). Importantly, it is likely that specific calcium influx and release pathways control distinct downstream targets that can have opposing effects on motility (Gomez and Zheng, 2006). However, since many MS ion channels are activated by multiple stimuli, manipulating mechanically activated currents specifically is challenging. For example, TRPC1 subunits are known to assemble into channels that are activated by both mechanical and chemical signals (Wu et al., 2010). Another major challenge to our understanding of mechanical signaling, is the composition and regulation of ECM adhesions. This is particularly difficult in growth cones, as point contact adhesions are smaller and more dynamic than focal adhesions found in non-neuronal cells. Proteomic based approaches are being attempted for focal adhesions, but are hampered by sample heterogeneity (Kuo et al., 2012; Humphries et al., 2015). Point contact adhesions will certainly also be highly heterogenous within growth cones and their composition is regulated by cross talk from guidance cue receptors to control mechanical forces (Myers et al., 2011; Gomez and Letourneau, 2014). In addition, point contact adhesions have not been observed *in vivo*, where they will clearly differ depending on substratum association. During axon pathfinding to distal targets, growth cones will bind many different adhesive substrata, ranging from ECM proteins to cell adhesion molecules on neighboring neurons and glia. It is important to both visualize and manipulate point contact adhesions *in vivo* to test their roles in axon guidance.

Determining the roles of tissue elasticity and mechanical signaling is particularly difficult *in vivo*. While it is clear that growth cones must migrate across widely varying elastic microenvironments en route to their proper synaptic targets, it is not known whether changes in tissue elasticity influences growth cone morphology or motility. For example, motoneuron axons begin in the soft CNS, but exit by penetrating the surrounding basal lamina (Santiago-Medina et al., 2015) to enter the sclerotome, which they preferentially cross within the rostral half (Keynes and Stern, 1984). After entering the periphery, MN axons are sorted toward targets in the body wall and limb muscles (Bonanomi and Pfaff, 2010), which are significantly more rigid tissues(Engler et al., 2004; Discher et al., 2009). Distinguishing specific effects of the mechanical environment vs. chemical signals is difficult *in vivo*. However, progress has been made by examining the effects of mutations in ECM cross linking enzymes that regulate the mechanical, but not the chemical composition of the environment (Kim et al., 2014). Additional functional studies should test the effects on axon guidance of manipulations that both increase and decrease tissue elasticity. Moreover, these functional studies can be coupled with direction measurements of elastic modulus within tissues *in vivo* using AFM (Franze, 2011).

## Conflict of Interest Statement

The authors declare that the research was conducted in the absence of any commercial or financial relationships that could be construed as a potential conflict of interest.
